# Comparing geriatric assessment tools for predicting negative health outcomes in older adults

**DOI:** 10.1186/s12916-026-05008-2

**Published:** 2026-07-09

**Authors:** Ahmad Abbadi, Francesco Innocenti, Giorgi Beridze, Emmanouil Kokoroskos, Alberto Zucchelli, Tobias Nordström, Caroline Wachtler, Laura Fratiglioni, Davide L. Vetrano, Amaia Calderón-Larrañaga

**Affiliations:** 1 Aging Research Center, Department of Neurobiology, Care Sciences and Society, Karolinska Institutet and Stockholm University, Solna, Sweden; 2 Department of Medical Epidemiology and Biostatistics, Karolinska Institutet, Solna, Sweden; 3 Unit for Analysis, Public Health Agency of Sweden, Stockholm, Sweden; 4 Division of Family Medicine and Primary Care, Department of Neurobiology, Care Sciences, and Society, Karolinska Institutet, Huddinge, Sweden; 5 Husläkarmottagning Täby Centrum, Lideta Mälardalen AB, Täby, Sweden; 6 Department of Clinical and Experimental Sciences, University of Brescia, Brescia, Italy; 7 Department of Clinical Sciences at Danderyds Hospital, Karolinska Institutet, Solna, Sweden; 8 Academic Primary Health Care Centre, Region Stockholm, Stockholm, Sweden; 9 Stockholm Gerontology Research Centre, Stockholm, Sweden

**Keywords:** Clinical decision-making, Geriatric assessment, Geriatric health care, Risk adjustment, Prospective cohort study

## Abstract

**Background:**

Amid global population aging, evidence-based geriatric assessment tools are essential for clinical decision-making and risk stratification. Despite growing interest, few studies have comprehensively compared the discriminative ability of existing tools, particularly as new tools have recently become available. In this study, we aimed to perform such a comparison across a wide range of patient-relevant health outcomes.

**Methods:**

This population-based prospective cohort study used data from the Swedish National study on Aging and Care in Kungsholmen (SNAC-K). We included 3,108 adults aged ≥ 60 years at baseline (2001–2004), who were followed up for up to six years. Seven tools (Health Assessment Tool [HAT], SNAC-K Frailty Index (FI) [SNACK-FI], Primary Care FI [PC-FI], Intrinsic Capacity [IC], Geriatric 8 [G8], Charlson Comorbidity Index [CCI], and Cumulative Illness Rating Scale [CIRS]) were evaluated in terms of their discriminative ability for formal care use, institutionalization, dementia, disability, injurious falls, self-rated health, quality of life, unplanned hospitalization, and mortality, using Harrell’s C-index estimated from unadjusted cause-specific Cox models.

**Results:**

HAT, IC, and SNACK-FI consistently ranked among the top three performers across all outcomes. The highest C-indices were observed for institutionalization (HAT 0.93 [0.91, 0.95], IC 0.93 [0.90, 0.94], SNACK-FI 0.92 [0.89, 0.94]); 1-year mortality (HAT 0.88 [0.85, 0.91], SNACK-FI 0.87 [0.84, 0.91], IC 0.86 [0.82, 0.89]); dementia (HAT 0.87 [0.85, 0.89], IC 0.88 [0.86, 0.90], SNACK-FI 0.86 [0.83, 0.88]); and formal care use (HAT 0.83 [0.81, 0.86], IC 0.85 [0.83, 0.88], SNACK-FI 0.80 [0.77, 0.83]). Tools incorporating physical function metrics (e.g., gait speed) showed higher discriminative ability than those that omitted them. IC and SNACK-FI showed marginal and clinically negligible occasional gains over HAT ( ≤ 0.02 differences in C-index), despite greater complexity and a larger number of indicators. Guideline-endorsed tools (e.g., G8, CCI, CIRS) showed comparatively lower discrimination across outcomes.

**Conclusions:**

Contemporary geriatric assessment tools show promise. Tools incorporating physical function metrics demonstrated superior discriminative ability, suggesting these measures may be integral to geriatric prognosis and risk stratification. Given the underperformance of several established tools, reappraisal of current guideline recommendations may be warranted.

## Background

The global demographic transition is marked by a significant increase in the aging population [1]. By 2050, an estimated 22% of the world’s population will be over the age of 60 [2]. In response to this shift, the World Health Organization (WHO) has championed the concept of healthy aging, building on the foundation of active aging [1]. The goal is not merely to extend lifespan, but to ensure that these additional years are lived in good health and quality of life [1, 3].

The complexity of health in older age, combined with non-linear aging trajectories, challenges healthcare professionals and risk stratification efforts [2]. Identifying candidates for proactive or preventive care—and communicating evidence-based decisions—requires careful judgment by clinicians and public health experts [4–6]. Although geriatric decision-making tools have proliferated [7, 8], it often remains unclear which tool to use and on what basis [8].

In this context, it is paramount to select tools that are theory-grounded, reliably predict health outcomes, and are feasible to implement in terms of cost and time, possibly through the use of routinely collected clinical data [1, 9]. Such tools must support efficient, clinically actionable decision-making [10] while ensuring high adoption and usability in healthcare and public health settings [11].

Amid these challenges, the Health Assessment Tool (HAT) has emerged as a comprehensive yet parsimonious geriatric assessment tool that balances discriminative ability with practical implementation [12]. Developed using five health indicators capturing physical and cognitive function, mild and severe disability, and chronic disease burden [12, 13], HAT has been internally [12, 14] and externally validated [15], has outperformed several frailty instruments in predicting mortality and healthcare use [16], and has been found to be feasible and acceptable for use in primary care by both older patients and healthcare providers [17].

Other tools proposed for primary care include the Intrinsic Capacity (IC), aligned with the WHO healthy aging framework by emphasizing functional reserves across multiple domains rather than deficit accumulation [1, 2, 9], and the Primary Care Frailty Index (PC-FI), a frailty index constructed exclusively from routinely collected electronic health record data to enhance applicability in primary care [18]. In research settings—with potential for translation into routine practice—the SNAC-K Frailty Index (SNACK-FI), conceptually similar to HAT, has shown high discriminative ability, and is derived from detailed clinical and functional assessments [19]. In secondary and tertiary care, guideline-recognized instruments include the Geriatric 8 (G8), widely used for oncology screening [20, 21]; the Cumulative Illness Rating Scale (CIRS), which provides a structured assessment of morbidity burden [22]; and the Charlson Comorbidity Index (CCI), a standard mortality risk index based on International Classification of Diseases (ICD) diagnostic codes [23].

These seven tools represent a broad spectrum of conceptual approaches to geriatric assessment;  ranging from deficit-accumulation and comorbidity-based indices to function- and capacity-oriented frameworks, while also capturing variation in data requirements and real-world feasibility across care settings.

Few studies have conducted direct, head-to-head comparisons of geriatric assessment tools in terms of their ability to predict key health outcomes or have considered emerging tools [16]. Moreover, the limited comparative research to date has disproportionately focused on healthcare utilization outcomes (e.g., unplanned admissions and emergency care use), while contemporary geriatric research recognizes that outcomes of importance extend beyond mortality to include independence and well-being, which older adults often highly prioritize [1, 2, 6, 9, 12, 18, 24, 25]. Accordingly, by spanning these domains, our study enables a multidimensional and patient-centered assessment of prognostic tool performance, addressing the limitations of prior research, which has typically focused on a narrower range of endpoints.

The aim of this study is to compare the HAT, IC, PC-FI, G8, CIRS, CCI, and SNACK-FI in terms of their discriminative ability across a wide range of patient- and public health-relevant outcomes to inform geriatric decision-making.

## Methods

### Study design, population, and data sources

This is a population-based prospective cohort study using data from the ongoing Swedish National study on Aging and Care in Kungsholmen (SNAC-K). In SNAC-K, individuals aged ≥ 60 years living in Kungsholmen, Stockholm, were randomly invited to participate [26, 27]. A total of 3,363 individuals accepted and participated in the baseline study visit between 2001-2004 [26, 27].

Participants are followed up in different intervals depending on their age; participants < 78 years are followed up every 6 years, and those aged ≥ 78 years every 3 years [26, 27]. Additional file 1: Figure S1 shows the follow-up frequency and timeline in SNAC-K [27]. Participants undergo comprehensive examinations by physicians, nurses, and psychologists, alongside questionnaires on physical, mental, and social health [26, 27]. Of the 3,363 baseline participants, 3,108 had sufficient data across the evaluated tools and were included.

### Geriatric assessment tools

This study evaluated seven geriatric assessment tools developed for use across different healthcare settings. The geriatric assessment tools were computed using baseline data. Further details on the computation of each tool are provided in Additional file 1.

#### Health assessment tool (HAT)

HAT integrates five health indicators, namely personal activities of daily living (P-ADL), instrumental activities of daily living (I-ADL), gait speed, cognition assessed through the Mini-Mental State Examination (MMSE), and number of chronic diseases [28], to generate an overall health status score ranging from 0 to 10 [12]. HAT was originally computed and validated using nominal response models, in which the health indicators were first categorized using data-driven thresholds and then regressed on a latent health variable [12, 15].

To enhance computability, a revised HAT was developed using a linear combination of its five indicators. Each indicator was normalized and rescaled to range from 0 (worst) to 1 (best), ensuring consistent directionality across domains. The normalized indicators were then combined using equal weights (0.2 per domain) and rescaled to range between 0-10. This approach enables direct comparability across indicators and preserves the interpretability of HAT on a 0–10 scale.

#### SNAC-K frailty index (SNACK-FI)

SNACK-FI is a multidimensional measure of frailty developed within the SNAC-K cohort using a data-driven deficit selection approach [19]. It includes 40 health deficits encompassing symptoms, signs, diseases, and disabilities, which were selected and weighted in an optimized way using a genetic algorithm to enhance discriminative ability and cohort specificity [19]. Scores range from 0 (best) to 1 (worst).

#### Primary care frailty index (PC-FI)

PC-FI was developed from SNACK-FI to support the early identification of frailty using exclusively primary health record data [18]. It was constructed from 25 health deficits derived from the ICD, Ninth Revision (ICD-9), ATC medication codes, and exemption codes, using a genetic optimization algorithm for all-cause mortality prediction [18]. The PC-FI was developed in a cohort of 308,280 Italian primary care patients aged ≥ 60 and externally validated in the SNAC-K study [18]. Scores range from 0 (best) to 1 (worst).

#### Intrinsic capacity (IC)

There is no clear consensus on how the IC should be operationalized [3]. Therefore, we proposed a pragmatic and easily computable approach using commonly collected geriatric indicators aligned with the WHO Integrated Care for Older People (ICOPE) framework [1, 2], which specifies five domains: cognition, measured through MMSE; vitality, measured using hand grip strength, appetite loss, and unintentional weight loss; locomotion, assessed using gait speed, balance, and lower limb strength; psychological well-being, assessed using the Montgomery–Åsberg Depression Rating Scale (MADRS); and sensory function, assessed using vision and hearing tests [29].

Each indicator was normalized and rescaled to range from 0 (worst) to 1 (best), ensuring consistent directionality across domains. Domain scores were constructed from the included indicators using equal weights. The normalized domain scores were then summed to yield a total score ranging between 0–10.

#### Geriatric 8 (G8)

G8 is a brief, oncology-established frailty screening tool originally developed and validated in older patients with cancer [20, 21]. It comprises eight items covering the following domains, operationalized following the scoring algorithm proposed by Soubeyran et al [21], with minor adaptations based on data availability in SNAC-K: nutrition, measured through appetite loss and unintentional weight loss; mobility, measured through ability to get in and out of bed to/from a chair and to handle personal care independently; neuropsychological status, assessed using depression and dementia diagnoses; body mass index; medication use, based on the total number of prescribed drugs; self-rated health; and age [20, 21].

The final G8 score was computed by summing all item scores to yield a total score ranging from 0 (worst) to 17 (best). A total score of 14 or below is considered abnormal and typically prompts referral for a comprehensive geriatric assessment [20, 30].

#### Charlson comorbidity index (CCI)

CCI was developed to predict 1-year mortality after hospital admission and is based on a set of ICD codes covering conditions such as myocardial infarction, chronic pulmonary disease, dementia, diabetes, liver disease, malignancies, and HIV/AIDS [23]. Over time, CCI has become widely used as a proxy for overall morbidity burden. [31, 32] In this study, the CCI coding algorithm developed by the Swedish National Board of Health and Welfare (Socialstyrelsen) [33] was used to create dichotomous variables indicating the presence or absence of each included condition. Scores can range from 0 to 37.

#### Cumulative Illness Rating Scale (CIRS)

CIRS was developed to provide a brief yet comprehensive and reliable assessment of morbidity burden [22]. It evaluates 13 functionally distinct domains grouped by body system (i.e., cardiac, respiratory, eye/ear/nose/throat, upper gastrointestinal, lower gastrointestinal, renal, genitourinary, musculoskeletal, central nervous system, dermatological, endocrine, haematological, and psychiatric) [22]. Each domain is rated on a five-point severity scale ranging from 0 (no impairment) to 4 (extremely severe impairment), yielding a theoretical total score from 0 to 52 [22].

### Outcomes

**Fig. 1:**
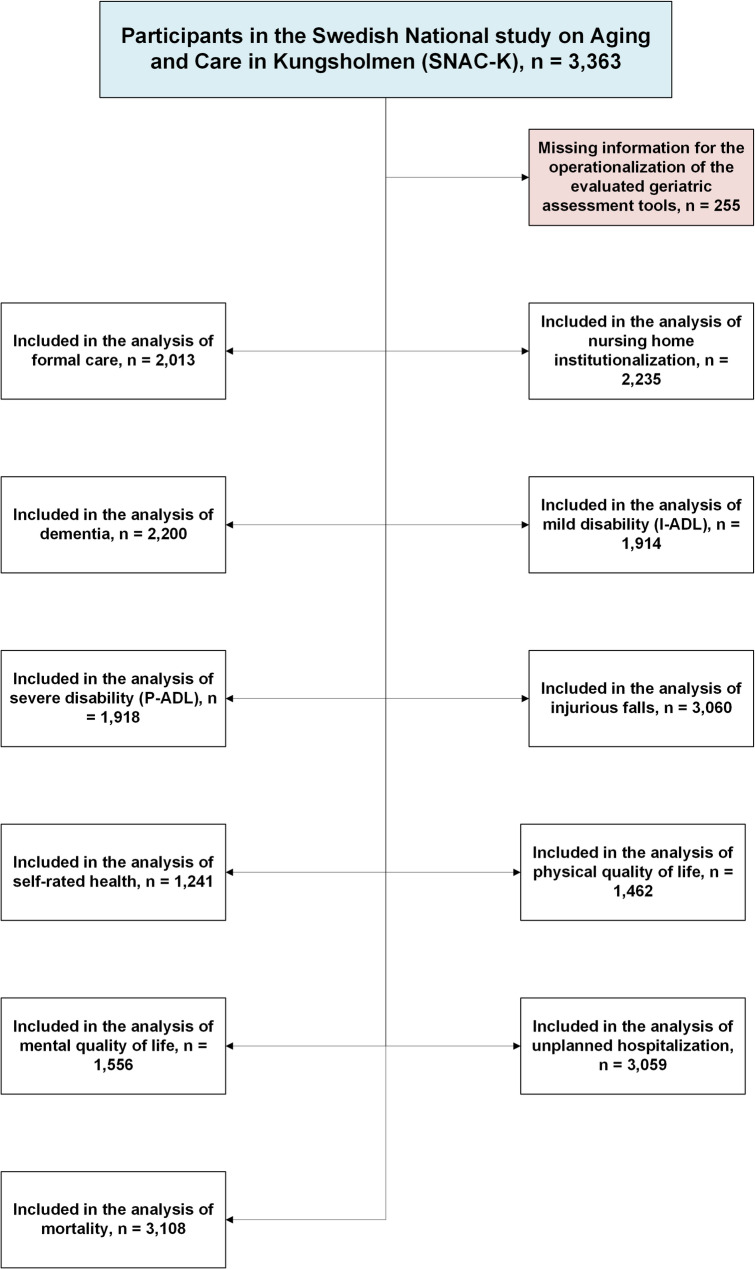
Flowchart of study inclusion for participants free from the outcomes at baseline

Eleven binary negative health outcomes were operationalized using SNAC-K follow-up data or national registers and were assessed over a 6-year follow-up period to ensure at least one SNAC-K follow-up assessment for all participants. Outcomes included formal care use, institutionalization, dementia, severe disability, mild disability, injurious falls, poor self-rated health, poor physical quality of life, poor mental quality of life, unplanned hospitalization, and all-cause mortality. Additionally, unplanned hospitalization and all-cause mortality were also examined over a 1-year follow-up period due to their clinical and public health relevance. Participants with the outcomes at baseline were excluded from the analysis of the respective outcome. Figure 1 shows the flowchart of study inclusion. Detailed information on how each outcome was defined and captured is available in Additional file 1.

### Statistical analysis

Descriptive statistics were reported as counts and percentages for categorical variables, and as means with standard deviations (SD) for continuous variables. The discriminative ability of each tool was assessed using Harrell’s C-index from unadjusted cause-specific Cox proportional hazards models, as our aim was to evaluate the intrinsic discriminative performance of each tool independently of other covariates. The models were corrected for censoring using Somers’ D rank statistic transformed to concordance (C) based on inverse hazard predictions [34]. Participants free of the outcome at baseline (index date) were followed until the occurrence of the event of interest or the end of follow-up (6 years; censoring date), whichever came first.

Additional information on the statistical, subgroup, and ancillary analyses, including the handling of missing data, is available in Additional file 1.

All analyses were performed using Stata version 17 (StataCorp LLC, College Station, TX, USA), and plots were generated in R version 4.4.2 (R Foundation for Statistical Computing, Vienna, Austria). The TRIPOD (Transparent Reporting of a multivariable prediction model for Individual Prognosis Or Diagnosis) guidelines [35] were followed and are available in Additional file 1.

## Results

Of the 3,108 study participants included in the study, 1,113 were male (35.8%). Overall, 33.6% had university-level education, while 49.4% high school education. The mean age at baseline was 74.2 (SD 11.0). Mean scores for the different geriatric assessment tools were: 6.7 (SD 2.2) for HAT (scale 0-10), 7.4 (SD 0.9) for IC (scale 0-10), 0.1 (SD 0.1) for SNACK-FI and PC-FI (scale 0–1), 14.1 (SD 1.7) for G8 (scale 0–17), 0.8 (SD 1.2) for CCI (scale 0–37), and 2.4 (SD 2.3) for CIRS (scale 0–52) (Table 1). Additional file 1: Table S1 shows the age-stratified baseline characteristics of the study population.

**Table 1:** Baseline characteristics of the study population

	Males		Females		Total	
**Age**, mean SD	71.6	10.0	75.6	11.2	74.2	11.0
**Sex (female)**, n %					1 995	64.2
**Civil status**, n %						
Married	724	65.0	646	32.5	1 370	44.2
Widower/Widow	122	11.0	691	34.7	813	26.2
Unmarried/Single	169	15.2	340	17.1	509	16.4
Divorced/Separated	98	8.8	313	15.7	411	13.2
Missing			5		5	
**Education**, n %						
Elementary	146	13.1	380	19.2	526	17.0
High school	470	42.2	1 059	53.4	1 529	49.4
University	497	44.7	544	27.4	1 041	33.6
Missing			12		12	
**Occupation**, n %						
Manual worker	189	17.1	535	27.2	724	23.6
Non-manual worker	918	82.9	1 429	72.8	2 347	76.4
Missing	6		31		37	
**P-ADL**, mean SD	0.1	0.5	0.2	0.9	0.2	0.8
**I-ADL**, mean SD	0.5	1.4	0.9	1.9	0.7	1.8
**MMSE**, mean SD	28.4	3.2	27.4	5.1	27.7	4.5
**Gait speed**, mean SD	1.1	0.4	0.9	0.5	1.0	0.5
**Count of chronic diseases**, mean SD	3.9	2.5	4.3	2.5	4.2	2.5
**HAT**, mean SD	7.3	1.9	6.4	2.4	6.7	2.2
**IC**^a^, mean SD	7.7	0.9	7.3	0.9	7.4	0.9
**SNACK-FI**, mean SD	0.1	0.1	0.1	0.1	0.1	0.1
**PC-FI**, mean SD	0.1	0.1	0.1	0.1	0.1	0.1
**G8**^a^, mean SD	14.3	1.6	14.0	1.7	14.1	1.7
**CCI**, mean SD	0.8	1.3	0.7	1.2	0.8	1.2
**CIRS**, mean SD	2.1	2.2	2.6	2.3	2.4	2.3

*n* number, *SD* standard deviation, *P-ADL* personal activities of daily living, *I-ADL* instrumental activities of daily living, *MMSE* mini-mental state examination, *HAT* health assessment tool, *IC* intrinsic capacity, *SNACK-FI* SNACK-frailty index, *PC-FI* primary care-frailty index, *G8* geriatric 8, *CCI* charlson comorbidity index, *CIRS* cumulative illness rating scale

^a^Prior to imputation

### Prediction of negative health outcomes

**Fig. 2:**
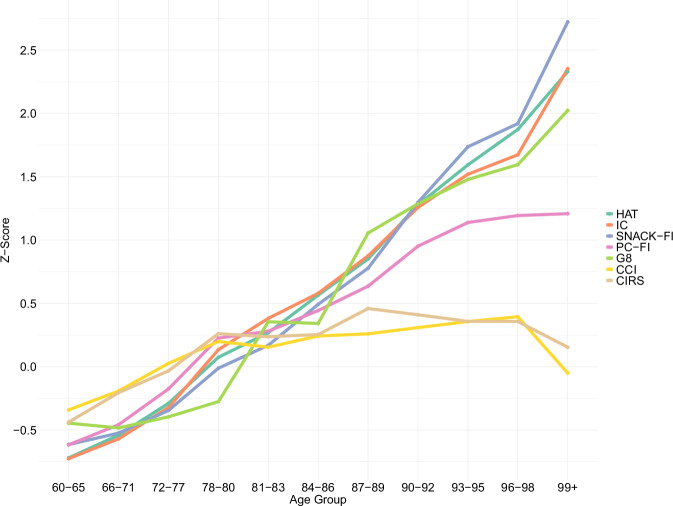
Distribution of standardized scores (i.e., z-scores) by age group and tool among SNAC-K participants

The standardized scores (i.e., z-scores) across age groups and tools are presented in Fig. 2. Scores increased with age across all tools. CCI, CIRS, and PC-FI appeared to plateau at older ages, with CCI and CIRS plateauing at earlier ages (≥ 78 years) than PC-FI ( ≥ 93 years). In contrast, HAT, SNACK-FI, IC, and G8 showed steadily increasing trends across age groups.

**Fig. 3:**
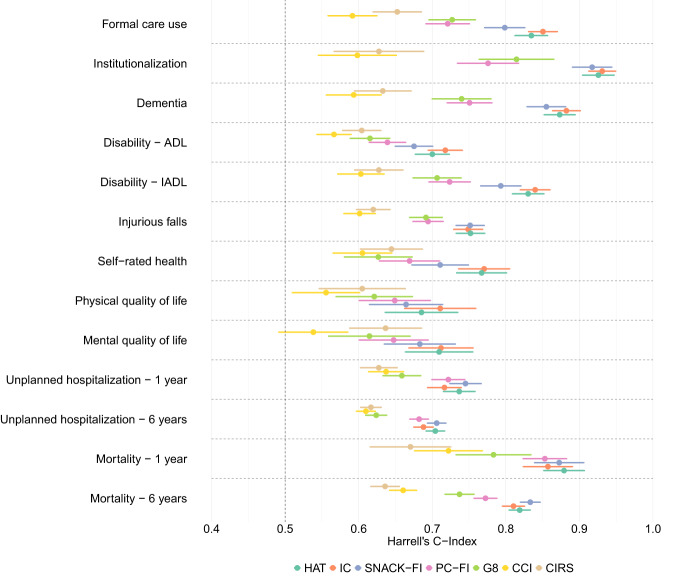
Discriminative ability of the seven tools across the 13 health outcomes

Figure 3 illustrates the discriminative ability of the seven tools across the 13 outcomes, and Additional file 1: Table S2 reports the corresponding C-index estimates and the number of events per outcome. For formal care use, IC performed best (C-index 0.85 [95% CI 0.81, 0.86]), followed by HAT (0.83 [0.81, 0.86]). Institutionalization was best predicted by HAT (C-index 0.93 [0.90, 0.95]), IC (0.93 [0.90, 0.94]), and SNACK-FI (0.92 [0.90, 0.94]). For incident dementia, IC performed best (0.88 [0.86, 0.90]), followed by HAT (0.87 [0.85, 0.89]) and SNACK-FI (0.86 [0.83, 0.88]).

Overall, severe disability was predicted better than mild disability. IC performed best (severe: 0.84 [0.82, 0.86]; mild: 0.71 [0.69, 0.74]), followed by HAT (severe: 0.83 [0.81, 0.86]; mild: 0.70 [0.68, 0.73]). Injurious falls were predicted equally by HAT, IC and SNACK-FI (0.75 [0.73, 0.77]). Poor self-rated health was best predicted by HAT (0.77 [0.73, 0.80]) and IC (0.77 [0.72, 0.80]).

For quality of life, SF-12 physical health was best predicted by IC (0.71 [0.66, 0.76]), followed by HAT (0.69 [0.64, 0.73]). In contrast, SF-12 mental health was best predicted by HAT and IC (0.71 [0.66, 0.76]). For 6-year unplanned hospitalization, SNACK-FI had the highest predictive performance (0.71 [0.69, 0.72]), followed by HAT (0.70 [0.69, 0.72]).

For mortality, discrimination was highest for HAT and SNACK-FI but varied by follow-up time; at 1 year, HAT performed best (0.88 [0.85, 0.91]), whereas at 6 years, SNACK-FI performed best (0.83, [0.82, 0.85]). The remaining tools showed lower discriminative ability across outcomes. Bootstrapped and optimism-corrected estimates for HAT, IC, and SNACK-FI are shown in Additional file 1: Table S3. Additional file 1: Figure S2 shows complete-case and imputed estimates for IC and G8, with higher discrimination for both tools in the imputed dataset.

The sensitivity and specificity of the tools based on data-driven cut-off points are shown in Table S4. Overall, HAT, IC, and SNACK-FI showed similar performance across outcomes, balancing good sensitivity and specificity. In contrast, PC-FI was consistently more sensitive across outcomes, whereas G8 and CCI were more specific at the data-driven Youden’s index cut-off points. To facilitate comparison and clinical usability, decision curve analyses are shown in Additional file 1: Figure S3 and S4: HAT and IC showed the highest net benefit across outcomes, while PC-FI was generally the third best-performing model.

Calibration curve plots are shown in Additional file 1: Figures S5–S17, displaying observed versus predicted risks for each tool and outcome. The discriminative performance of the current and previous HAT operationalizations was nearly identical, with the linear approach providing marginal improvements in prediction alongside substantial gains in computational efficiency (Additional file 1: Figure S18). The distribution of individual indicators is presented in Additional file 1: Table S5, and Additional file 1: Figure S19 shows density and box plots for the current and previous HAT versions.

### Sex and age stratification

**Fig. 4:**
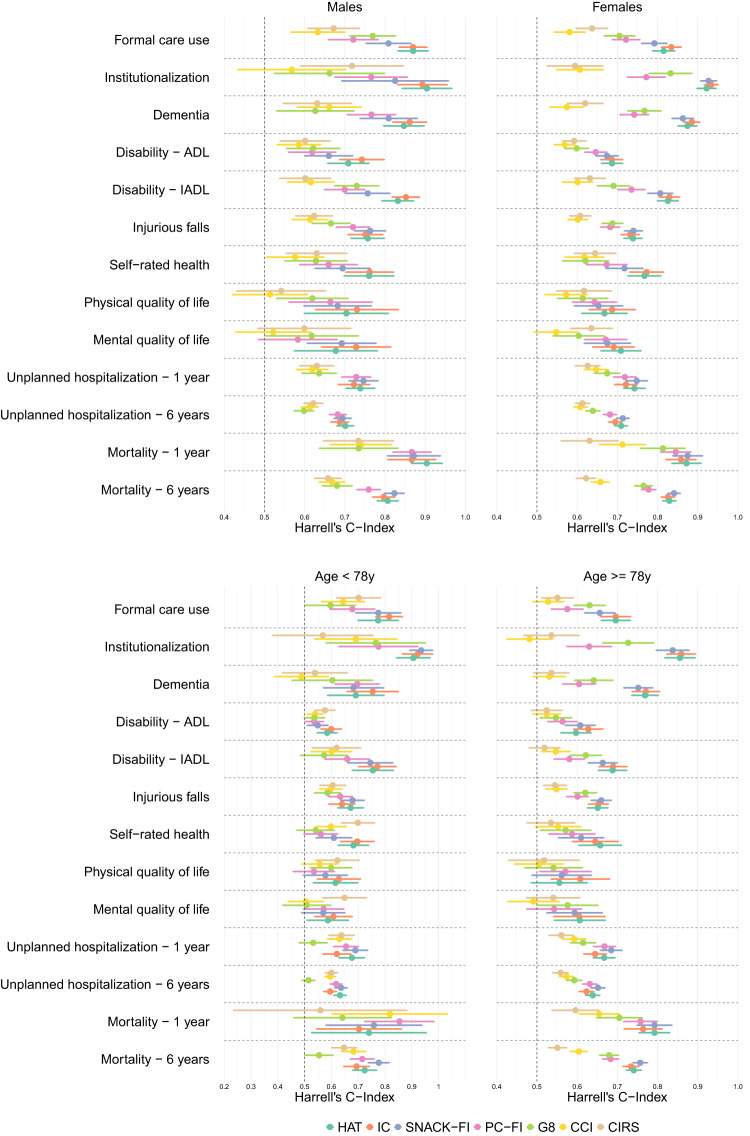
Stratification of the discriminative ability of the seven tools across the 13 health outcomes by sex and age

HAT and IC consistently ranked among the top two performing tools across all outcomes and across sex and age strata (Fig. 4). SNACK-FI performed comparably in most strata, although it performed slightly less well among males. In contrast, the performance of G8, CCI, and CIRS was substantially lower among adults aged < 78 years.

## Discussion

In this population-based study comparing several geriatric assessment tools used across levels of care, HAT, IC, and SNACK-FI consistently ranked among the top three performers across outcomes. IC and SNACK-FI showed negligible gains over HAT ( ≤ 0.02 differences in C-index) despite greater data collection burden and a higher number of indicators, and these differences are unlikely to be clinically meaningful for individual risk stratification or decision-making. A key strength shared by these high-performing tools is their emphasis on physical function—particularly gait speed—, which is a well-established clinical predictor of a wide range of adverse outcomes in older adults [1, 2, 36]. Tool performance was consistent across sexes but tended to decline among younger-old adults, likely reflecting functional resilience and heterogeneity of this group [15, 16].

Selecting geriatric assessment tools requires balancing discriminative ability with real-world feasibility, including indicator burden, administrative complexity, and required clinical expertise [37]. While discrimination is a key metric [37], optimal tool design favors parsimonious, informative, and minimally intercorrelated indicators [38]. Our analyses showed that tools incorporating objective physical function metrics, such as gait speed, substantially outperformed those that omitted this domain [20, 21]. Furthermore, including an excessive number of indicators may introduce stochastic “noise”, which can paradoxically weaken prognostic accuracy [39]. However, the discriminative ability varied across the 13 outcomes. These differences may reflect both the underlying constructs captured by each instrument and the outcome-specific baseline risk profiles of the SNAC-K study population.

HAT’s parsimonious architecture—incorporating only five indicators—achieved discriminative ability comparable to, and in some cases exceeding, that of more complex instruments such as IC or SNACK-FI, which include a larger number of indicators. These negligible gains are unlikely to translate into meaningful clinical or public health utility and may be offset by the increased administrative burden and computational time associated with more complex tools. Moreover, our findings challenge the assumption that comprehensive inclusion of all WHO-defined IC domains is necessary to optimize discriminative ability or adequately capture health in an aging population [1, 2]. While theoretically robust, IC offers a broad overview of domains that may warrant intervention and supports a holistic view of older adults’ health [1–3, 9]. However, certain IC components may contribute little to prognostic accuracy in practice, suggesting that tool development should prioritize empirically validated domains over exhaustive theoretical coverage. Additionally, the computation of IC remains subject to debate [9]. While we propose a pragmatic and highly predictive approach in this study, the use of different measures to operationalize the domains of IC may result in differences in discriminative ability despite their theoretical overlap. Nonetheless, these additional indicators in IC and SNACK-FI might assist clinicians in guiding more individualized interventions.

Assessing frailty and multidimensional health status remains central to therapeutic planning and intervention stratification [5]. Nevertheless, many existing tools fall short in capturing longitudinal health trajectories, including treatment response, the effects of preventive interventions, or changes in quality of life. This underscores the need for rigorous validation of prognostic instruments prior to clinical adoption [40]. Our study adds to this multidisciplinary effort by showing that geriatric assessment provides clinically practical tools with public health value that can support decision-making. Moreover, newer tools may further enhance risk stratification, including in healthier populations. Together, these advances can help bridge the gap between geriatric care and other disciplines by enabling the use of reliable, accurate, validated—yet simple—tools to support high-quality care, identify older adults who may benefit from geriatric-led multidimensional assessment, and improve access to appropriate services.

While some of the tools examined in this study are endorsed by clinical guidelines and professional bodies to inform decision-making [2, 3, 25, 41, 42], our findings suggest that their utility may be context dependent. For example, despite recommendations by the European Association of Urology to use tools such as G8, CIRS, CCI, and the Clinical Frailty Scale (CFS) to guide treatment decisions—based on their presumed ability to predict mortality and, by extension, life expectancy [41, 42]—our analyses indicate that these instruments show reduced discriminative ability for mortality and unplanned hospitalization in a community-dwelling population that is broadly representative of older adults, compared with the potentially sicker populations typically seen in secondary or tertiary care. Although CFS is widely advocated in clinical practice [24, 43], its reliance on clinical or patient judgment [44] may contribute to variability and suboptimal prognostic accuracy [45, 46].

Furthermore, the optimal balance between sensitivity and specificity is inherently context-dependent, varying by clinical setting and the nature of the subsequent intervention [47, 48]. In primary care, where the goal is often to identify individuals for preventive or mitigating interventions through geriatric evaluation, high sensitivity may be prioritized to minimize false negatives [48]. Conversely, in tertiary care settings, where determining eligibility for major surgery or complex medical regimens is often central, high specificity may be favored to reduce the risk of post-therapeutic complications among falsely identified candidates [48]. Although HAT, IC, and SNACK-FI showed reasonable sensitivity and specificity when using exploratory, data-driven cut-offs derived from Youden’s index, these thresholds are study-specific, have not been externally validated, and are not optimized to maximize the applicability of each tool in real-world settings. Optimal cut-offs are inherently context-dependent and should be tailored to the clinical setting and intended intervention rather than applied universally. Tailoring cut-off points to distinct healthcare priorities could enhance clinical utility by aligning tool performance with setting-specific requirements. In decision curve analyses, HAT and IC offered the highest net benefit across all outcomes. HAT, in particular, reconciles prognostic accuracy with implementability through its streamlined design. Future work should prioritize integrating these tools into routine workflows, particularly to support the early identification of older adults who may benefit from further assessment or targeted interventions.

Promising avenues include deploying assessment approaches that rely on non-clinical personnel or leveraging artificial intelligence (AI)-enabled mobile health technologies [49, 50]. These innovations could facilitate self-administered or remotely supervised evaluations of cognitive function (e.g., digital adaptations of the MMSE) and physical performance (e.g., gait speed measurement with artifact correction). Evidence supports the viability of AI-driven applications for capturing essential health domains with minimal supervision—particularly in resource-constrained settings—while maintaining predictive performance and clinical utility [49, 50].

### Strengths and limitations

The study has several strengths. It uses prospective cohort data from SNAC-K, which, from its inception, has featured comprehensive and well-documented data collection conducted by physicians, nurses, and psychologists. This enabled the comparison of multiple geriatric assessment tools over a long follow-up period and across a wide range of clinically relevant outcomes. The tools selected for analysis are commonly used in both clinical practice and research, enhancing the relevance and translational value of the findings. Moreover, sex- and age-stratified analyses allowed evaluation of tool performance across subpopulations. Although socioeconomic inequalities in health persist, Sweden’s tax-funded universal health system aims to ensure that access to healthcare and institutional care is based on health needs rather than socioeconomic status [51].

However, several limitations should be acknowledged. First, participants in the SNAC-K cohort tend to be healthier, more educated, and wealthier than the general Swedish population of the same age, which may limit generalizability. This may lead to lower event rates and influence the observed discriminative performance of the tools. Second, some tools lacked complete baseline data, requiring imputation of missing indicators. These tools (IC and G8) were evaluated in both complete-case and imputed analyses, with better performance observed in the imputed dataset. Nevertheless, this improvement did not alter their ranking relative to the other tools, suggesting that differences in predictive performance between indices are driven more by the constructs they capture than by data availability. In addition, the CIRS tool and the quality-of-life outcomes were each missing one indicator required for proper operationalization. Third, three of the assessed tools were developed or operationalized by members of the research team using data from the SNAC-K cohort. HAT and SNACK-FI were developed by the research team, while IC was operationalized using a new algorithm. PC-FI was not developed using data from SNAC-K, although members of the research team were involved in its development using Italian data. While HAT was externally validated [15], the SNACK-FI and IC require external validation. Fourth, some outcomes may have been affected by competing risk due to attrition, as participants may have died or dropped out before subsequent assessments. Fifth, cut-off points were derived using a data-driven approach, which might not reflect clinically meaningful thresholds or optimize performance across all outcomes. Sixth, sensitivity and specificity alone may be insufficient for clinical and public health decision-making compared with positive and negative predictive values. Seventh, not all tools were originally designed to assess the full range of outcomes included in this study, nor have they been validated across all levels of care (screening, primary, secondary, and tertiary settings). Nevertheless, this comparative assessment provides valuable insights into the potential utility of these tools for clinical and public health decision-making across diverse healthcare contexts.

## Conclusions

As health systems confront the increasing demands of an aging population, pragmatic and theory-grounded geriatric assessment tools offer a critical pathway toward more personalized, efficient, and evidence-informed decision-making. In this context, selecting tools that balance discriminative ability with clinical feasibility is essential. In our study, HAT, IC, and SNACK-FI—each emphasizing physical function—consistently ranked among the top performers across outcomes. Notably, the simplified computation and streamlined design of HAT enhance its applicability in routine clinical care. Future research should focus on scalable approaches to implementing these tools, including evaluation across diverse levels of care and the development of digital or self-administered formats, to broaden accessibility and maximize their impact across healthcare settings.

## Supplementary information

Supplementary material: Additional file 1: Supplementary methods, Tables S1–S5, Figures S1–S19, TRIPOD Checklist

## Data Availability

The datasets generated and/or analyzed during the current study are not publicly available due to ethical and data-sharing restrictions and applicable legislation, including the General Data Protection Regulation (GDPR). However, access to the original data is available to the research community upon approval by the SNAC-K Coordination Group. Applications for data access can be submitted via the SNAC-K website (https://www.snac-k.se/for-researchers/application-form/).

## Abbreviations

AI: artificial intelligence
C: concordance
CCI: Charlson Comorbidity Index
CFS: Clinical Frailty Scale
CI: confidence interval
CIRS: Cumulative Illness Rating Scale
G8: Geriatric 8
HAT: Health Assessment Tool
I-ADL: instrumental activities of daily living
IC: Intrinsic Capacity
ICOPE: Integrated Care for Older People
MADRS: Montgomery–Åsberg Depression Rating Scale
MMSE: Mini-Mental State Examination
P-ADL: personal activities of daily living
PC-FI: Primary Care Frailty Index
SD: standard deviation
SNAC-K: Swedish National study on Aging and Care in Kungsholmen
SNACK-FI: SNAC-K Frailty Index
TRIPOD: Transparent Reporting of a multivariable prediction model for Individual Prognosis Or Diagnosis
WHO: World Health Organization
