# Supplementary material for: Comparing geriatric assessment tools for predicting negative health outcomes in older adults
Source: BMC Med. 2026 Jul 9;24:379. doi: 10.1186/s12916-026-05008-2 (PMC13348859; doi:10.1186/s12916-026-05008-2)
Supplement: Supplementary file 1 — Supplementary material: Additional file 1: Supplementary methods, Tables S1–S5, Figures S1–S19, TRIPOD Checklist [file 12916_2026_5008_MOESM1_ESM.docx]

**Table 1: Baseline characteristics of the study population**

|  | **Males** | | **Females** | | **Total** | |
| --- | --- | --- | --- | --- | --- | --- |
| **Age**, mean SD | 71.6 | 10.0 | 75.6 | 11.2 | 74.2 | 11.0 |
| **Sex (female)**, n % |  |  |  |  | 1 995 | 64.2 |
| **Civil status**, n % |  |  |  |  |  |  |
| Married | 724 | 65.0 | 646 | 32.5 | 1 370 | 44.2 |
| Widower/Widow | 122 | 11.0 | 691 | 34.7 | 813 | 26.2 |
| Unmarried/Single | 169 | 15.2 | 340 | 17.1 | 509 | 16.4 |
| Divorced/Separated | 98 | 8.8 | 313 | 15.7 | 411 | 13.2 |
| Missing |  |  | 5 |  | 5 |  |
| **Education**, n % |  |  |  |  |  |  |
| Elementary | 146 | 13.1 | 380 | 19.2 | 526 | 17.0 |
| High school | 470 | 42.2 | 1 059 | 53.4 | 1 529 | 49.4 |
| University | 497 | 44.7 | 544 | 27.4 | 1 041 | 33.6 |
| Missing |  |  | 12 |  | 12 |  |
| **Occupation**, n % |  |  |  |  |  |  |
| Manual worker | 189 | 17.1 | 535 | 27.2 | 724 | 23.6 |
| Non-manual worker | 918 | 82.9 | 1 429 | 72.8 | 2 347 | 76.4 |
| Missing | 6 |  | 31 |  | 37 |  |
| **P-ADL**, mean SD | 0.1 | 0.5 | 0.2 | 0.9 | 0.2 | 0.8 |
| **I-ADL**, mean SD | 0.5 | 1.4 | 0.9 | 1.9 | 0.7 | 1.8 |
| **MMSE**, mean SD | 28.4 | 3.2 | 27.4 | 5.1 | 27.7 | 4.5 |
| **Gait speed**, mean SD | 1.1 | 0.4 | 0.9 | 0.5 | 1.0 | 0.5 |
| **Count of chronic diseases**, mean SD | 3.9 | 2.5 | 4.3 | 2.5 | 4.2 | 2.5 |
| **HAT**, mean SD | 7.3 | 1.9 | 6.4 | 2.4 | 6.7 | 2.2 |
| **IC***, mean SD | 7.7 | 0.9 | 7.3 | 0.9 | 7.4 | 0.9 |
| **SNACK-FI**, mean SD | 0.1 | 0.1 | 0.1 | 0.1 | 0.1 | 0.1 |
| **PC-FI**, mean SD | 0.1 | 0.1 | 0.1 | 0.1 | 0.1 | 0.1 |
| **G8***, mean SD | 14.3 | 1.6 | 14.0 | 1.7 | 14.1 | 1.7 |
| **CCI**, mean SD | 0.8 | 1.3 | 0.7 | 1.2 | 0.8 | 1.2 |
| **CIRS**, mean SD | 2.1 | 2.2 | 2.6 | 2.3 | 2.4 | 2.3 |
| *Prior to imputation  n: number, SD: standard deviation, P-ADL: personal activities of daily living, I-ADL: instrumental activities of daily living, MMSE: mini-mental state examination, HAT: Health Assessment Tool, IC: Intrinsic Capacity, SNACK-FI: Study of Aging and Care in Kungsholmen-Frailty Index, PC-FI: Primary care- Frailty index, G8: Geriatric 8, CCI: Charlson Co-morbidity Index, CIRS: Cumulative Illness Rating Scale | | | | | | |
